# Karyotype diversity and chromosomal organization of repetitive DNA in *Tityus obscurus* (Scorpiones, Buthidae)

**DOI:** 10.1186/s12863-017-0494-6

**Published:** 2017-04-17

**Authors:** Bruno Rafael Ribeiro de Almeida, Susana Suely Rodrigues Milhomem-Paixão, Renata Coelho Rodrigues Noronha, Cleusa Yoshiko Nagamachi, Marlyson Jeremias Rodrigues da Costa, Pedro Pereira de Oliveira Pardal, Johne Souza Coelho, Julio Cesar Pieczarka

**Affiliations:** 1 Laboratório de Citogenética, Centro de Estudos Avançados da Biodiversidade, Instituto de Ciências Biológicas, Universidade Federal do Pará, Avenida Augusto Corrêa, n°01, Av. Perimetral, s/n. Guamá, 66075-900 Belém, Pará Brazil; 2 Instituto Federal de Educação, Ciência e Tecnologia de Goiás, Campus Valparaíso de Goiás, BR-040, km 6, Avenida Saia Velha, S/N, Área 8, Parque Esplanada V, 72876-601 Valparaíso de Goiás, Goiás Brazil; 3 Laboratório de Entomologia Médica e Artrópodes Peçonhentos, Núcleo de Medicina Tropical, Universidade Federal do Pará, Avenida Generalíssimo Deodoro, 92, 66055-240 Belém, Pará Brazil

**Keywords:** Holocentric chromosomes, Repetitive DNA, Multivalent association, Scorpiones, *Tityus*

## Abstract

**Background:**

Holocentric chromosomes occur in approximately 750 species of eukaryotes. Among them, the genus *Tityus* (Scorpiones, Buthidae) has a labile karyotype that shows complex multivalent associations during male meiosis. Thus, taking advantage of the excellent model provided by the Buthidae scorpions, here we analyzed the chromosomal distribution of several repetitive DNA classes on the holocentric chromosomes of different populations of the species *Tityus obscurus* Gervais, 1843, highlighting their involvement in the karyotypic differences found among them.

**Results:**

This species shows inter- and intrapopulational karyotype variation, with seven distinct cytotypes: A (2n = 16), B (2n = 14), C (2n = 13), D (2n = 13), E (2n = 12), F (2n = 12) and G (2n = 11). Furthermore, exhibits achiasmatic male meiosis and lacks heteromorphic sex chromosomes. Trivalent and quadrivalent meiotic associations were found in some cytotypes. In them, 45S rDNAs were found in the terminal portions of two pairs, while TTAGG repeats were found only at the end of the chromosomes. In the cytotype A (2n = 16), the U2 snRNA gene mapped to pair 1, while the H3 histone cluster and *C*_*0*_ *t*-1 DNA fraction was terminally distributed on all pairs. *Mariner* transposons were found throughout the chromosomes, with the exception of one individual of cytotype A (2n = 16), in which it was concentrated in heterochromatic regions.

**Conclusions:**

Chromosomal variability found in *T. obscurus* are due to rearrangements of the type fusion/fission and reciprocal translocations in heterozygous. These karyotype differences follow a geographical pattern and may be contributing to reproductive isolation between populations analyzed. Our results also demonstrate high mobility of histone H3 genes. In contrast, other multigene families (45S rDNA and U2 snRNA) have conserved distribution among individuals. The accumulation of repetitive sequences in distal regions of *T. obscurus* chromosomes, suggests that end of chromosome are not covered by the kinetochore.

## Background

Holocentric chromosomes, which occur in approximately 750 species of eukaryotes, are characterized by the presence of a diffuse centromere wherein the kinetochore proteins are located along the chromatids [1]. The holocentric condition appears to favor the emergence of extensive intra- and interspecific karyotype variability mainly generated by chromosomal fusions or fissions [2, 3]. This feature has emerged independently during evolution, especially among plants, nematodes and arthropods [4]. In Arachnida, holocentric chromosomes are found in the Aranae, Acari and Scorpiones. Within the latter order, they have been reported only in the Buthidae [5].

Buthidae scorpions offer an excellent model for studying the dynamics of holocentric chromosomes, since the diploid number is highly conserved in some genera but highly variable in others [6]. Moreover, some members show complex multivalent pairing during male meiosis [7], reflecting the gradual accumulation of multiple fusions/fissions and/or translocations [8]. Depending on the degree of structural heterozygosity, the chains or rings may differ in size at the interpopulational, intrapopulation, and/or intraindividual levels [9–11].

In Buthidae, few studies have examined the chromosomal distribution of repetitive DNA, and are restricted to the 45S rDNA and telomeric sequences mapping [11, 12]. In other arthropods with holocentric systems, repetitive sequences have proven useful in the chromosomal mapping-based identification of chromosome pairs [13]. Such sequences also function in homologous meiotic pairing [14], chromosomal segregation during cell division [15], and the stability of chromosomal ends in the absence of telomerase [16]. Thus, additional studies are warranted to improve our understanding of the dynamics of the holocentric chromosomes in Scorpiones.

The genus *Tityus* (Scorpiones, Buthidae) comprises some 170 species that are exclusively neotropical and occur from the Dominican Republic to Central Argentina [17]. Only 10% of the species in this genus have been cytogenetically analyzed; they exhibit karyotypes with low diploid numbers (2n = 5 to 27) and no heteromorphic sex chromosomes [18]. Thus, taking advantage of the excellent model provided by the Buthidae scorpions, here we analyzed the chromosomal distribution of several repetitive DNA classes on the holocentric chromosomes of different populations of the species *Tityus obscurus* Gervais, 1843, highlighting their involvement in the karyotypic differences found among them.

## Results

### Karyotype

The karyotype of *T. obscurus* consists of holocentric chromosomes and lacks heteromorphic sex chromosomes. The diploid number and chromosome size showed inter- and intrapopulation variations, with seven distinct cytotypes.

**Fig. 1:**
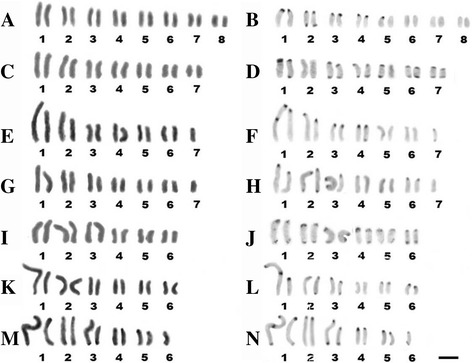
Karyotypes of *Tityus obscurus* with giemsa stain (**a**, **c**, **e**, **g**, **i**, **k**) and C-banded (**b**, **d**, **f**, **h**, **j**, **l**, **n**): (**a**, **b**) cytotype A, 2n = 16. **c**, **d** cytotype B, 2n = 14. **e**, **f** cytotype C, 2n = 13. **g**, **h** cytotype D, 2n = 13. **i**, **j** cytotype E, 2n = 12. **k**, **l** cytotype F, 2n = 12. **m**, **n** cytotype G, 2n = 11. Barr = 10 μm

The cytotype A (2n = 16) comprises twenty one individuals from Belém, Santa Bárbara, Moju, Benevides, Bragança and Acará, with karyotype constituted by two large and fourteen medium chromosomes (Fig. 1a). C-bands were observed on terminal regions of pairs 1, 2, 3, 4 and 5, while the pairs 6, 7 and 8 showed no visible C-bands (Fig. 1b).

The cytotype B (2n = 14) comprises one individual male from Santarém and five individuals from Afuá, with karyotype constituted by four large and ten medium chromosome (Fig. 1c). C-bands were observed on terminal regions of pairs 1, 3, 4 5, 6 and 7 while the pair 2 showed no visible C-bands (Fig. 1d).

The cytotype C (2n = 13) comprises one individual male from Afuá, with karyotype constituted by one extremely large, three large and nine medium chromosome (Fig. 1e). The pair 7 showed absence of homologous member. C-bands were observed on terminal regions of pairs 1, 2, 3, 4 and 7, while one interstitial C-band was visualized only on a homologous member of the pair 1 (Fig. 1f).

The cytotype D (2n = 13) comprises one individual male from Santarém, with karyotype constituted by five large and eigth medium chromosome (Fig. 1g). C-bands were observed on terminal regions of pairs 1, 2, 3 and 6 (Fig. 1h).

The cytotype E (2n = 12) comprises three individuals from Rurópolis and one individual male from Santarém, with karyotype constituted by six large and six medium chromosome (Fig. 1i). C-bands were observed on terminal regions of all pairs (Fig. 1j).

The cytotype F (2n = 12) comprises one individual male from Santarém, with karyotype constituted by one extremely large, five large and six medium chromosome (Fig. 1k). C-bands were observed on terminal regions of pairs 1, 2, 3, 5 and 6 while one interstitial C-band was visualized only on a homologous member of the pair 1 (Fig. 1l).

The cytotype G (2n = 11) comprises one individual male from Santarém, with karyotype constituted by one extremely large, five large and five medium chromosome (Fig. 1m). C-bands were observed on terminal regions of all pairs, while one interstitial C-band was visualized only on a homologous member of the pair 1 (Fig. 1n).

### Meiotic analysis

**Fig. 2:**
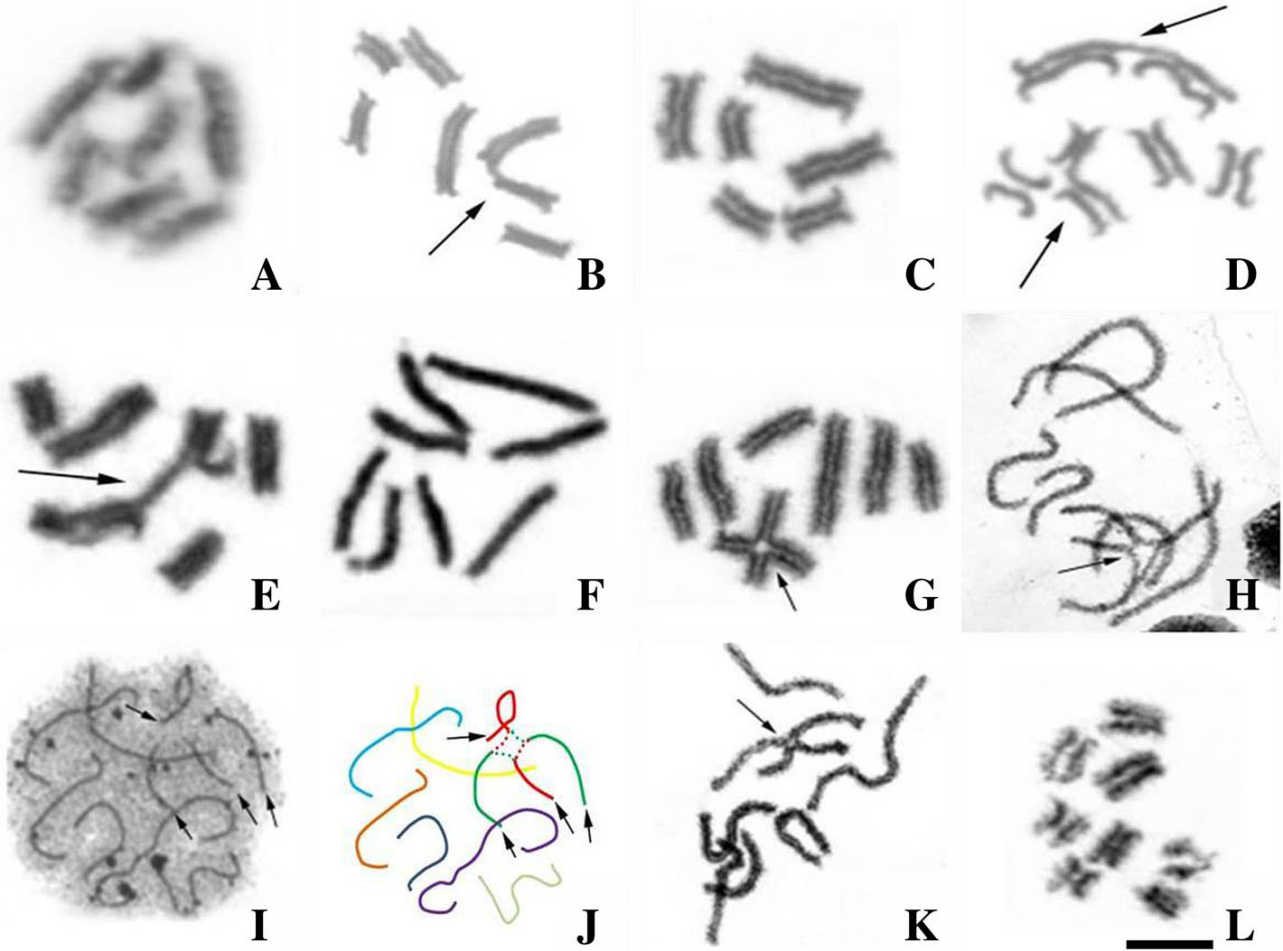
Meiotic configuration on different specimens of *T. obscurus*. **a** Cytotype B, with seven bivalents. **b** Cytotype C and D, with one trivalent and five bivalents. **c** Cytotype E, with six bivalents. **d** Cytotype F with three bivalents and two trivalent. **e** Cytotype G with four bivalents and one trivalent. **f** Cytotype A with eight bivalents. **g** Cytotype A with six bivalents and one quadrivalent. **h** Cytotype A with quadrivalent at beginning of pachytene (arrows points the asynaptic region). **i** Synaptonemal complex analysis in an early pachytene of cytotype A with quadrivalent; the *arrow points* the asynaptic region of the quadrivalent. **j** An schematic interpretation of Fig. 2I. **k** Cytotype A with quadrivalent at end of pachytene; note the full pairing of the quadrivalente. **l** Metaphase II, with eight chromosomes. *Arrows* in (**b**) (**d**) and (**e**) point trivalent associations. Barr = 10 μm

In meiotic cells, we did not observe chiasma during the post-pachytene phases. Different meiotic configurations were observed as follows: cytotype B (2n = 14) had seven bivalents (Fig. 2a); cytotypes C and D (both 2n = 13) had five bivalents and one trivalent (Fig. 2b); cytotype E (2n = 12) had six bivalents (Fig. 2c); cytotype F (2n = 12) had three bivalents and two trivalents (Fig. 2d); cytotype G (2n = 11) had four bivalents and one trivalent (Fig. 2e); most of the individuals of the cytotype A (2n = 16) had eight bivalents (Fig. 2f), while one specimen from Acará was heterozygous for a reciprocal translocation, and showed six bivalents and one quadrivalent (Fig. 2g).

Conventional staining and synaptonemal complex (SC) analysis in the specimen of the cytotype A (2n =16) from Acará, which was heterozygous for a reciprocal translocation, demonstrated that the central region of the quadrivalent was non-synaptic in initial and intermediate pachytene cells (Fig. 2h-j). The full pairing happened only at the end of the pachytene phase (Fig. 2k). In contrast, the bivalents were completely paired at the initial stage of pachytene (see Fig. 2h). After metaphase II, the cells of the analyzed individual were n = 8 (Fig. 2l).

### Chromosomal mapping of repetitive DNA

**Fig. 3:**
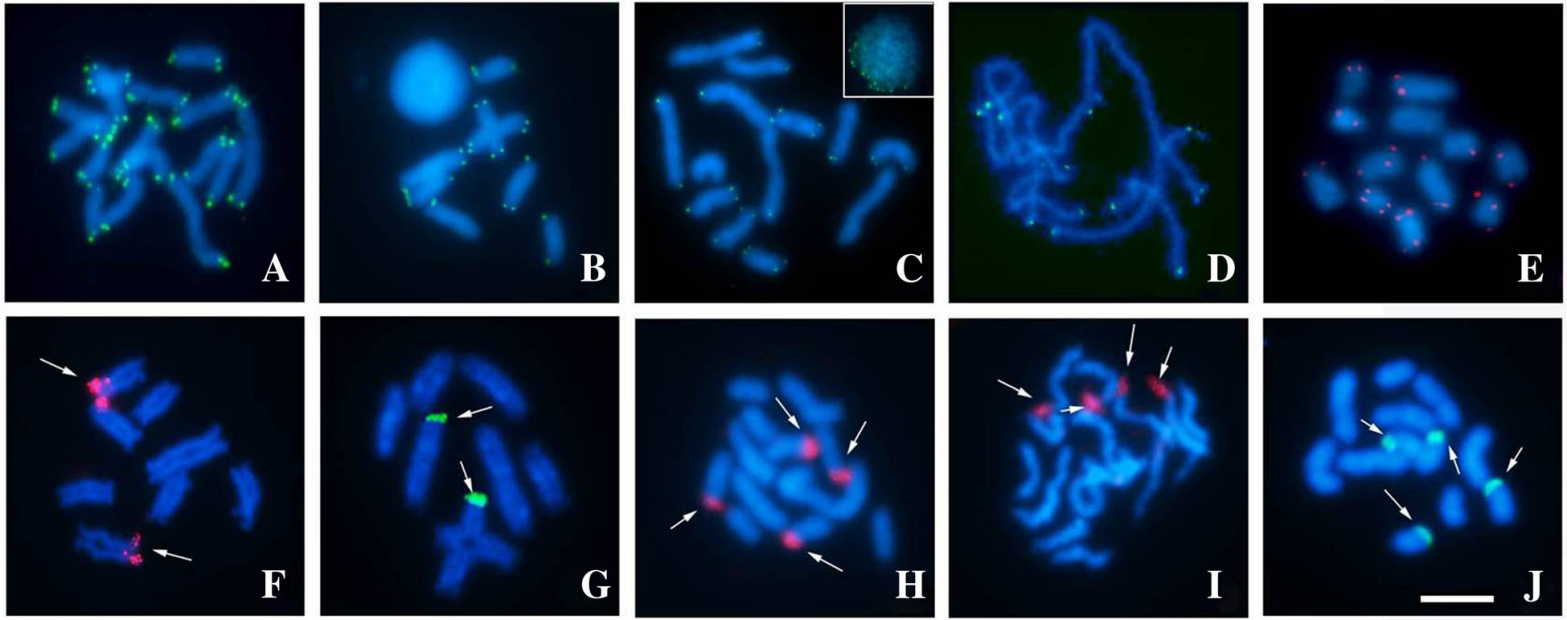
FISH with TTAGG (**a**-**e**) and 45S rDNA (**f**-**j**) probes in mitotic (**a**, **c**, **e**, **h**, **i**, **j**) and meiotic (**b**, **d**, **f**, **g**) chromosomes. **a**, **f** cytotype A. **b**, **g** Post-pachytene in cytotype A, a translocation bearer. **c**, **d**, **h** Cytotype F; the insert in (**c**) shows a nucleus with telomeres polarized to a single region; in (**d**) pachytene on the same sample in 3C. **e** Cytotype C (**f**) Cytotype E. The *arrows* in (**f**-**j**) point ribosomal sites. Barr = 10 μm

Mapping FISH of the TTAGG repeats was realized in cytotypes A (2n =16), E (2n = 12) and F (2n =12) and were restricted to the ends of all chromosomes (Fig. 3a-e). Analysis of the spatial distribution of this sequence in prophasic nuclei showed that the chromosomes were organized in a polarized configuration at the beginning of meiosis I (Fig. 3c).

In the cytotypes A (2n =16), C (2n =13) and F (2n = 12) the 45S rDNA was found in terminal regions in two pairs (Fig. 3f-j). In the specimen of the cytotype A, heterozygous for a reciprocal translocation, one of the pairs bore this sequence (pair 7) and was part of the quadrivalent observed during meiosis (Fig. 3g).

**Fig. 4:**
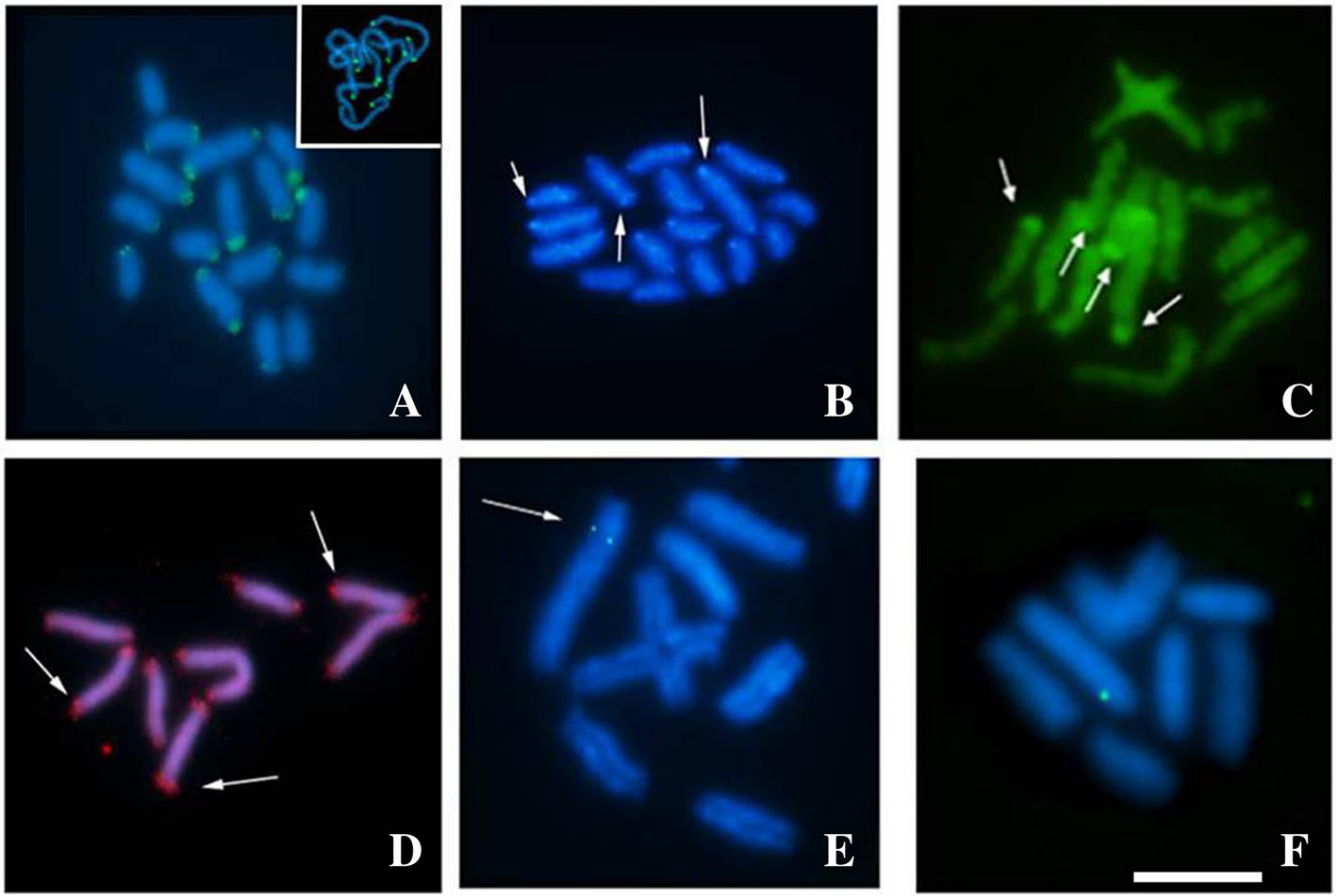
Heterochromatin and multigenic family mapping in 2n = 16 specimens. **a** *Cot*-1 DNA. The insert in (**a**) shows a meiotic cell in pachytene. **b** DAPI staining. **c** CMA_3_ staining. **d** FISH H3 histone probe showing terminal hybridization. **e** U2 snRNA gene maping in a 2n = 16 specimen, a translocation bearer. **f** U2 snRNA gene in a 2n =16 specimen with eight bivalents. Barr = 10 μm

In the cytotype A (2n = 16), chromosomal mapping of the *C*_*0*_ *t*-1 DNA fraction showed that the constitutive heterochromatin regions were rich in highly repetitive DNA sequences (Fig. 4a). In this cytotype, the DAPI+ bands coincided with the heterochromatic block, indicating that the CH is rich in AT base pairs (Fig. 4b). In contrast, CMA3+ bands were visualized at the terminal regions of four chromosomes (Fig. 4c). In addition, in the cytotype A, Histone H3 gene clusters were found in the terminal regions of all chromosomes (Fig. 4d), and the multigene family encoding the U2 snRNA was found on chromosome 1 (Fig. 4e, f).

**Fig. 5:**
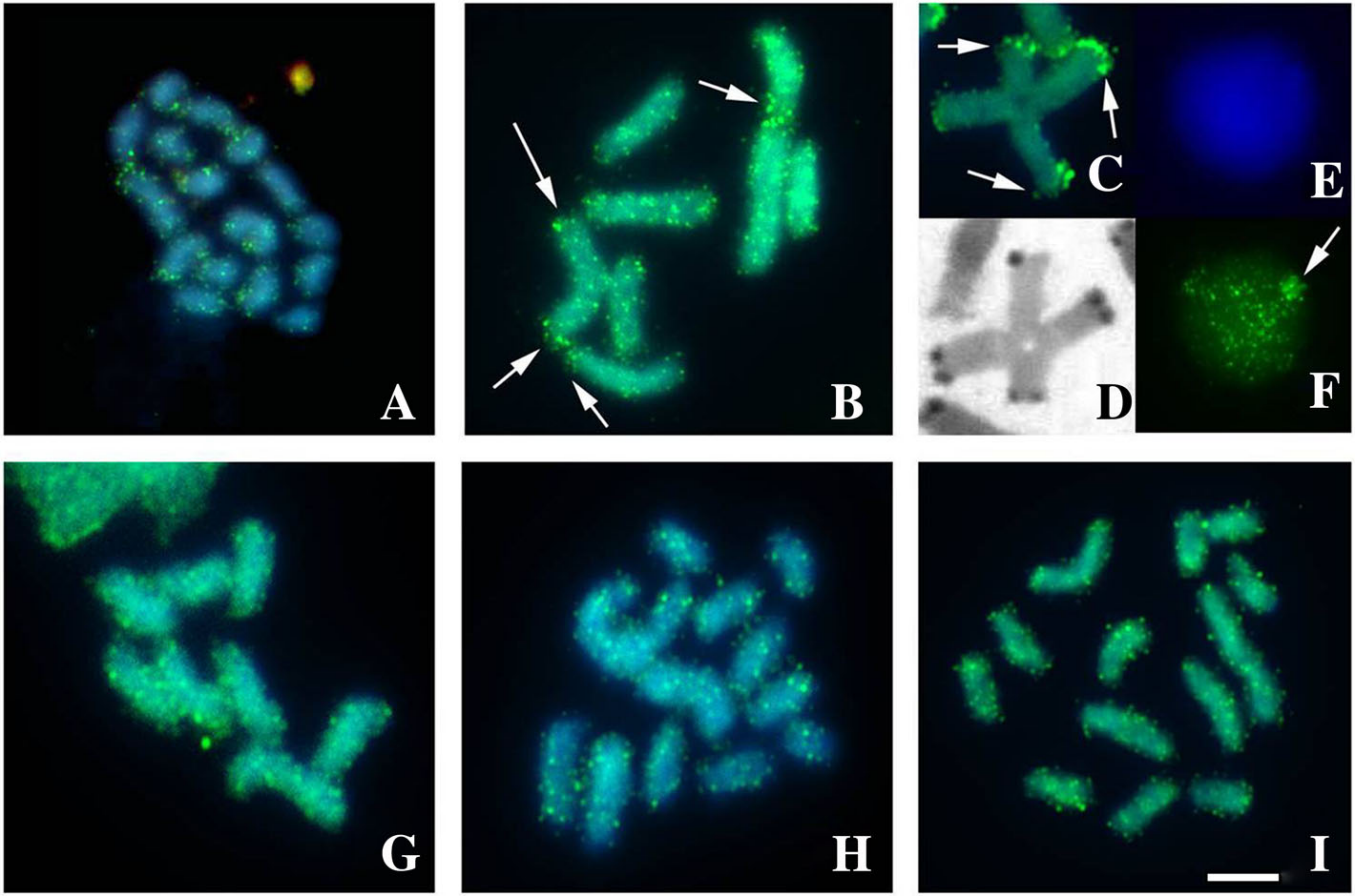
*Mariner* mapping in *T. obscurus*. **a**-**f** 2n = 16, a translocation bearer. **a** Mitotic metaphase. **b** Post-pachytene. **c** and **d** Post-pachytene after FISH and C-banding, with *Mariner* distribution in the constitutive heterochromatin of quadrivalent. **e** and **f** Interphase nucleus with packed distribution of *Mariner*. **g** Metaphase I of 2n = 16 specimen with eight bivalents. **h** 2n = 12, a translocation bearer. **i** 2n = 13, a translocation bearer. Barr = 10 μm

Regarding the *Mariner* transposon, the specimen of the cytotype A (2n =16), that was heterozygous for a reciprocal translocation, showed a large concentration of this transposon in the CH regions of some of its bivalents and in the quadrivalent (Fig. 5a-f). Others individuals of the cytotypes A (2n =16), C (2n =13) and F (2n = 12) had small clusters distributed along their chromosomes, either uniformly occupying euchromatic regions or as heterochromatin (Fig. 5g-i).

## Discussion

### The role of heterozygous rearrangements in chromosomal evolution of *Tityus obscurus*

Our date demonstrated than fusions/fissions are involved in the karyotype variability of *T. obscurus*. Several studies have identified polymorphisms of the diploid numbers within *Tityus* genus, resulting of these rearrangements. In the scorpion *Tityus bahiensis*, for example, the diploid number was found to differ across populations of southeastern Brazil, from 2n = 5 to 19 [19]. This occurs because the mitotic spindles of holocentric system join along the entire length of each chromatid, promoting normal segregation of both fused and fragmented chromosomes during anaphase [20]. In this study, fusions/fissions were found in heterozygosis among specimens of the cytotypes C (2n = 13), D (2n = 13), F (2n = 12) and G (2n = 11). In addition, we speculate that chromosome 1 of the cytotypes C, F and G may reflect a fusion, based on its extremely-large size and its involvement in trivalent associations during meiosis. The presence of an interstitial C-band would reinforce this hypothesis, as previously suggested for other organisms [21, 22]. Interestingly, this interstitial C-band was present on extremely large chromosome 1 of three *T. obscurus* cytotypes, and may be required to stabilize chromosomes resulting from fusion in this species. Absence of interstitial telomeric sites (ITSs) in the chromosome 1 of cytotype F (2n = 12) can be explained by the small size of the ITS due to the molecular erosion of repetitive sequences, escaping FISH resolution [23] or deletion of telomeres in the original chromosomes [24].

**Fig. 6:**
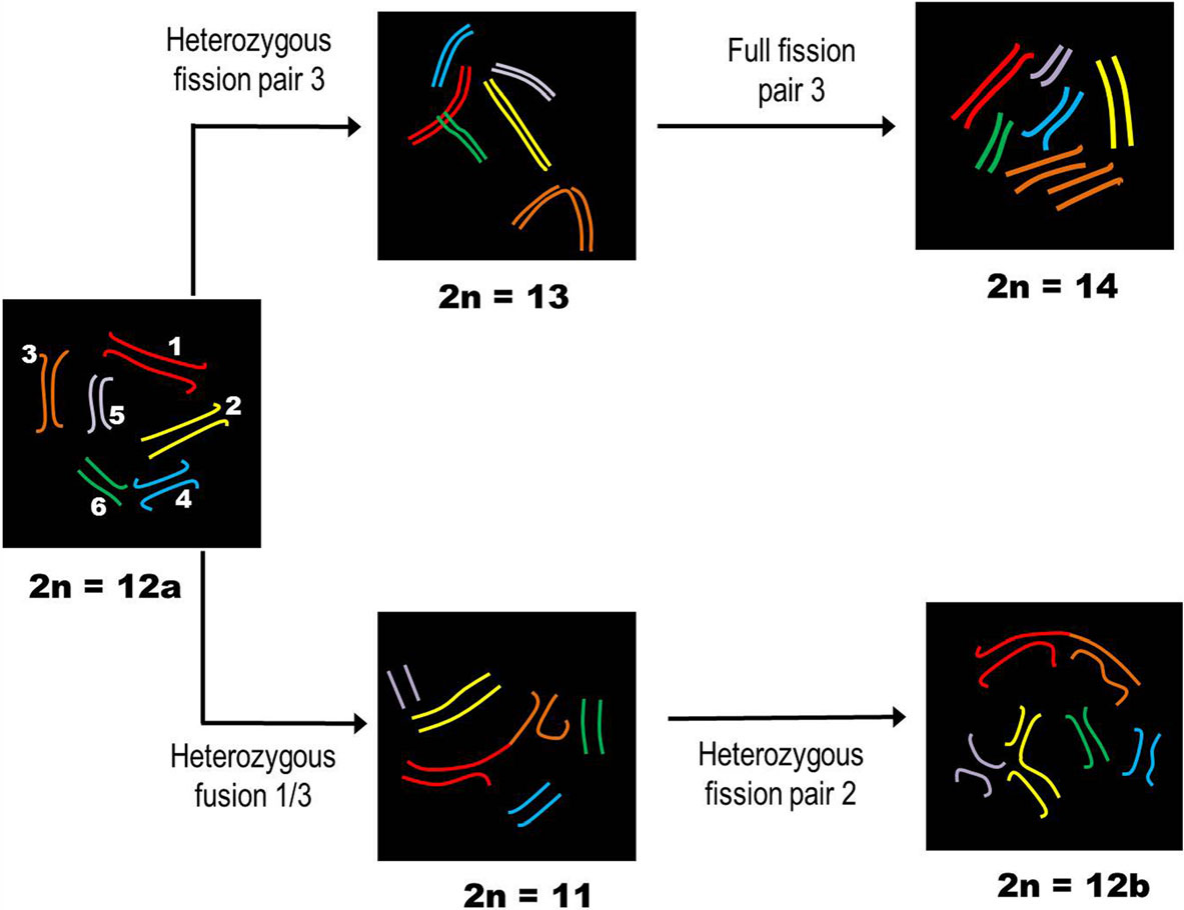
Schematic interpretation of karyotype evolution hypothesis proposed to specimens of *Tityus obscurus* from Santarém, Brazil

The Fig. 6 shows a schematic interpretation of the karyotype evolution of cytotypes of *T. obscurus* from Santarém. We proposed that the cytotype E (2n = 12) represent the ancestral karyotype of this population, and that fission events led to the formation of cytotypes C (2n = 13) and B (2n = 14), whereas the heterozygous fusion of pairs 1 and 3 of ancestral karyotype would have led to the appearance of the cytotype G (2n = 11), which would later originated the cytotype F through a heterozygous fission of par 2 (2n = 12). The visualization of TTAGG telomerics repeats only at the end of all chromosomes of cytotype F (2n = 12) suggests that even in those in which fission occurs, there may a telomere reconstitution system. This fact has been previously demonstrated for other taxa bearing holocentric chromosomes, such as Aphids and plants, in which telomerase acts constitutively in the reconstitution of telomeres in fragmented chromosomes [23, 25].

Repetitive sequence mapping enabled us to identify the chromosome pairs involved in the reciprocal translocation observed in a male of cytotype A (2n = 16) from Acará. The first pair was defined by exclusion; both pairs 1 and 2 are large and similar in size. The U2 snRNA gene, which maps to pair 1, was not included in the translocation, enabling us to conclude that pair 2 is involved in this rearrangement. The other pair was found to bear a 45S rDNA cluster, and thus corresponded to chromosome 7. Fertility was not affected by this translocation, since 100% of the cells in metaphase II had the appropriate haploid number (n = 8), indicating the presence of alternate chromosome segregation. The region between the breakpoints in the quadrivalent was non-synaptic during pachytene. Schneider et al. [26] also observed this in meiotic cells of four Buthidae, and proposed that it reflected a lack of homology between the rearranged segments. Similar phenomena have been observed in humans [27], pigs [28], boars [29], and in the grasshopper *Eyprepocnemis plorans* [30].

**Fig. 7:**
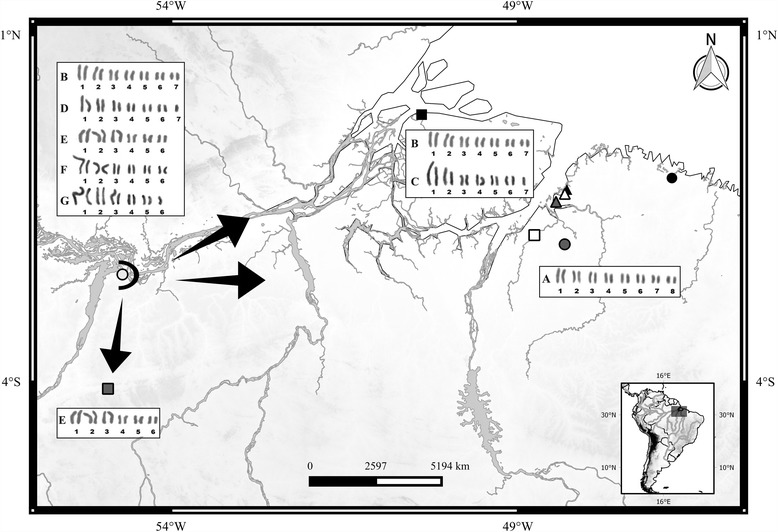
Collection places of individuals of *T. obscurus* and geographic distribution of the cytotypes*:* Santarém (*white circle*); Belém (*grey triangle*); Benevides (*white triangle*); Santa Bárbara (*black triangle*); Áfua (*black square*); Moju (*white square*); Acará (*grey circle*); Bragança (*black circle*); Rurópolis (*grey square*). The letters adjacent to the karyotypes represent their cytotypes. Note the greater diversity of karyotypes in Santarém (putative distribution center for the species). The *arrows* next Santarém indicate the dispersion of these cytotypes to marginal regions. This map is the work of Bruno Rafael Ribeiro de Almeida and used with permission

Our analysis of the geographic distribution of various *T. obscurus* karyotypes suggests that there is a central-marginal variation pattern (Fig. 7). According to the model of this kind of variation, populations in the center of a species geographic distribution have a higher genetic diversity than those at the margins [31, 32]. This model was observed in the chromosome inversions patterns of *Drosophila willistoni*, [33], *Trimerotropis pallidipennis* [34], and *Chironomus plumosus* [35], and in fusions of *Dichroplus pratensis* [36]. In the present work, the Santarém population would be central, while the others (Afuá, Rurópolis, Belém, Moju, Acará, Bragança, Santa Bárbara and Benevides) would be marginal.

### Genomic organization of repetitive DNA in holokinetics chromosomes of *Tityus obscurus*

In monocentric chromosomes, the centromere is usually surrounded by pericentromeric heterochromatin. It has been proposed that transcriptional inactivity of heterochromatin is required for competence of the centromere and/or that heterochromatin permits centromeric chromatin assembly, perhaps by facilitating expansion of CenH3 domains over flanking regions [37]. Satellite DNAs and transposable elements are main sequences found in the centromeric region [38, 39]. Our result of the mapping of the *C*_*0*_ *t*-1 DNA fraction suggests *a priori* that in *Tityus obscurus* there is no presence of highly repetitive DNAs associated with centromere domains, since the hybridization signals were observed only in the terminal regions of the meiotic and mitotic chromosomes. This hypothesis is supported by the fact that in most of the animals and plants bearing holocentric chromosomes the presence of centromeric repetitive DNAs was not observed [40]. The only exceptions to this patterns are two species of *Rhynchospora* (Cyperaceae) for which the centromeric satellite DNA *Tyba* was described [41]. In *Luzula elegans*, although cenH3 domains are distributed along the chromosomes, satellite DNAs and retrotransposons did not show expected distribution for wide centromere [42]; in addition, the sequencing of the genome of *Luzula elegans* and *C. elegans* showed absence of centromere-associated sequences [41, 43]; in the latter, the incorporation of cenH3 is independent of specific sequences [43]. In insects, absence of centromeric repetitive DNA, may be related to loss of cenH3 protein in holocentrics clades of insects [44].

In *Tityus obscurus* the presence of two pairs of homologues bearing of the 45S rDNA in the terminal region differed from the pattern observed in the other Buthidae, which present ribosomal clusters in one pair of homologues [11, 12]. Some authors have proposed that the NOR terminal position is necessary for the stability and correct segregation of holokinetic chromosomes during cell division, once decondensed NORs constrictions could promote an interruption in the kinetochore plate [45]. However, this model does not apply to buthid scorpions *Androctonus* genus, which present interstitial NORs and normal chromosome segregation [6].

The gene encoding Histone H3 was distally distributed in all chromosomes of cytotype A (2n = 16) of *T. obscurus*. Dispersed distributions, such as that observed in *T. obscurus*, have been found on monocentric chromosomes of grasshoppers [46, 47] and in the fish *Rachycentron canadum* [48]. These studies suggest that the dispersion of Histone H3 genes can occur by ectopic recombination, transposable elements or circular DNA [46]. The evolutionary significance of dispersed organization of Histone H3 in *T. obscurus* is not known; in achiasmatics organisms, such male of *Drosophila* sp. and females of Lepidoptera, rDNAs and telomeric repeats, respectively, act in recognition and pairing of homologs chromosomes during meiosis [14, 49]. In *Luzula elegans*, terminal satellites DNA form chromatin bridges between homologous chromosomes to ensure correct segregation during inverted meiosis [50]. In *T. obscurus* clusters Histone H3 can perform similar function.

This is the first study mapping the *Mariner* transposon in Arachnida. In our sample, this transposon was either dispersed throughout the euchromatin (in most specimens) or packed in the heterochromatin (in the 2n = 16 translocation bearer). The chromosomal distribution of this transposable element is extremely variable among organisms. In beetles, for example, *Mariner* was found only in heterochromatin [51], while in grasshoppers, it was reportedly restricted to euchromatin [52]. TEs tend to accumulate in genomic regions that have low recombination rates, such as pericentromeric heterochromatin, B chromosomes and sex chromosomes [53–55]. This pattern reflects a strong selection pressure against the deleterious effects of recombination in these regions [55]. The presence of *Mariner* in the euchromatin of *T. obscurus* can be partly explained by the absence of recombination during male meiosis, which is achiasmatic [19].

Repetitive sequences were concentrated at the terminal regions, as shown by our analyses of C-banding, telomeric sequences, *Cot*-1 DNA, 45S rDNA, H3 histone genes, and (in the 2n = 16 individual heterozygous for the translocation) *Mariner* transposons, while an expressed multigene family (U2 snRNA) mapped to an interstitial region. Our results are consistent with previous reports that repetitive sequences tend to be found in holocentric chromosomes terminals [56, 57], while single genes are always distributed throughout the genome [58–60]. Thus, the regions where the repetitive sequence accumulates (here, the terminal regions in *T. obscurus*) must somehow differ from the rest of the genome. While the kinetochore was believed to extend along the poleward face of the metaphase chromosome in holocentric chromosomes [61], Heckmann et al. [45] showed that this is not necessarily true, as the centromeric regions of *Luzula elegans* extend along most of the chromosome, but do not cover the terminal regions. Benavente [62] performed an ultrastructural analysis of chromosome of scorpion *Tityus bahiensis* and demonstrated that they are only partially covered by kinetochore. Based on our present results, we propose that the chromosomes of *T. obscurus* are not entirely holocentric; instead, we believe that the distal regions, with their accumulations of repetitive sequences, flanking the kinetochore, but are not covered by it.

## Conclusions

Chromosomal variability found in *T. obscurus* is due to rearrangements of the type fusion/fission and reciprocal translocations in heterozygous. These karyotype differences follow a geographical pattern and may be contributing to reproductive isolation between the analyzed populations. Our results also demonstrate high mobility of histone H3 genes that can act in recognition, pairing and segregation of homologue chromosomes. In contrast, other multigene families (45S rDNA and U2 snRNA) have conserved distribution among individuals, and showed to be reliable markers to elucidate chromosomal rearrangements in *T. obscurus. C*_*0*_ *t*-1 DNA fraction is terminally distributed on all pairs in *T. obscurus,* suggesting the absence of repetitive DNA associated with the wide centromere*.* The accumulation of repetitive sequences in distal regions of *T. obscurus* chromosomes suggests that distal portions of chromosome are not covered by the kinetochore.

## Methods

### Sample

Adult *Tityus obscurus* were captured in municipalities of Pará State, Brazil. Information on sex and collection sites of the individuals is described in Table 1 and Fig. 7). The specimens were deposited in the collection of Medical Entomology Laboratory and Venomous Arthropods of Tropical Medicine Center of UFPA.

**Table 1:** Details on the sample used in this study

N	Locality	Geographical coordinate
1 male/3 females	Belém, PA, Brasil	1°24′16.29″S/48°27′12.29″O
1 female	Santa Bárbara, PA, Brasil	1°13′40.15″S/48°17′46.53″O
2 males/10 females	Benevides, PA, Brasil	1°17′23.33″S/48°19′35.69″O
1 female	Moju, PA, Brasil	1°53′06.64″S/48°45′55.52″O
2 males	Acará, PA, Brasil	2°01′03.79″S/48°1910.42″O
1 male	Bragança, PA, Brasil	1°03′41.83″S/46°46′58.84″O
2 males/4 females	Afuá, PA, Brasil	0°08′51.08″S/50°23′24.25″O
5 males/2 females	Santarém, PA, Brasil	2°27′04.27″S/54°42′04.63″O
2 male/1 female	Rurópolis, PA, Brasil	4°06′02.68″S/54°54′34.65″O

### Karyotype analysis

Chromosome preparations were generated as described by [19]. Gonads were submitted to hypotonic treatment with 0.075 M KCl for 20 min and then fixed in acetic acid: methanol solution (3: 1 ratio) overnight. Fragments of gonads were macerated in 60% acetic acid, and cell suspension was spread on slides; after drying at 45 °C, chromosomes were stained with Giemsa 5%. Chromosomal measurements were performed through MicroMeasure software version 3.3 for Windows [63], and classified according to size in extremely large (EL), large (L), medium (M) and small (S). The karyotypes were organized using the Adobe Photoshop version 4.0 software. C-banding [64] and fluorochrome banding using 4,6-diamidino-2-phenylindole (DAPI) [65] and Chromomycin A3 (CMA_3_) [66] were performed according to the mentioned references.

### Synaptonemal complex analisys

Synaptonemal complex spreading was obtained as described by [67] with modifications. Gonads were maintained in Hanks solution for 10 min. Then they were macerated in 100 mM sucrose solution with the aid of needles. The cell suspension was spread on slides previously coated with paraformaldehyde 2% and incubated in humid chamber for 2 h at room temperature. After this, slides were washed in 0,08% Photo-flo solution for five minutes and stored at −80 °C. For visualization of the synaptonemic complex, slides were stained with a 50% silver nitrate solution, according to [68].

### Probes

The genomic DNA of one *T. obscurus* female was extracted with GenEluteMammalian Genomic DNA Miniprep Kit (Sigma–Aldrich, St. Louis, MO, USA), and was used as template for isolation of repetitive sequences. The telomeric motif (TTAGG) was amplified by PCR using the (TTAGG)_5_ and (CCTAA)_5_ complementary primers [69]. The genes encoding H3 histones and U2 snRNA were isolated as described by [70] using the primers: 5′-ATG GCT CGT ACC AAG CAG AC(ACG) GC-3′ and 5′-ATA TCC TT(AG) GGC AT(AG) AT(AG) GTG AC-3′ for H3 Histone; 5′-TCT CGG CCT (AT)(AT)T GGC TAA-3′ and 5′-G(AC)G GTA (GC)TG CAA TAC CGG-3′ for U2 snRNA. *Mariner* transposon partial sequence was obtained as described by [71] using primers MAR-188 F (5′-ATC TGR AGC TAT AAA TCA CT-3′) e MAR-251R (5-CAA AGA TGT CCT TGG GTG TG-3′). The PCR products were directed used as probes for FISH, after being analysed in agarose gel 1%. To construct the 45S rDNA probe, we used the pTa71 plasmid, which contains the genes 5.8S, 18S, 28S and their respective intergenic spacers, isolated from genome of *Triticum aestivum* [72].

The *C*_*0*_ *t*-1 DNA was obtained according to [73]. Initially, 500 μL of genomic DNA of *Tityus obscurus* (400 ng/μL at NaCl 0,3 M) were autoclaved for 3 to 5 min, and then denatured at 95 °C for 10 min. DNA was treated for 1 min with the enzyme S1 Nuclease, and subsequently frozen in liquid nitrogen. Afterwards it was precipitated using phenol/chloroform and ice-cold absolute ethanol, and kept in the freezer at −80 °C for 30 min. Then, the DNA was resuspended in ultrapure water and stored at −20 °C until use.

Probes were labeled by nick translation according to the kit available. All probes were labeled with digoxigenin-14-dUTP (Roche, Mannheim, Germany). Alternatively, some telomeric, Histone H3 and rDNA probes were labeled with biotin-11-dATP (Invitrogen, San Diego, CA, USA).

## FISH

Fluorescence *in situ* Hybridization (FISH) was performed as described by [74] with modifications. Briefly, the slides were incubated in RNAse and Pepsin solution for 1 h and 15 min, respectively, both at 37 °C. After, slides were fixed in 4% paraformaldehyde and dehydrated in ethanol series (70, 90 and 100%). Hybridization solution containing probes was denatured to 100 °C, spread on the slides, which were covered with coverslips. Chromosomal DNA was denatured to 70 °C. Hybridization occurred at 37 °C, overnight. Probes were visualized with Avidin-Cy3 or Anti-Digoxigenin-FITC. Chromosomes were counterstained with DAPI (Vectashield; Vector, Burlingame, California, USA).

## Abbreviations

*C*_*0*_*t*-1 DNA: DNA enriched for highly and moderately repetitive DNA sequences
CH: Constitutive heterocromatin
CMA_3_: Chromomycin A3
DAPI: 4,6-diamidino-2-phenylindole
PCR: Polymerase chain reaction
SC: Synaptonemal complex
snRNA gene: Small nuclear RNA gene
